# Honorary Degrees in Dentistry,—Why Not?

**Published:** 1899-07

**Authors:** 


### Domestic Correspondence.

### HONORARY DEGREES IN DENTISTRY,—WHY NOT?

To the Editor:

Sir,—Does it not strike you as absurd that, while the distinguished universities of Europe and America confer honorary degrees upon men who have earned them by their public services, either in their own specialty or some collateral branch, the National Association of Dental Faculties places unnatural obstacles in the way of the same honors being conferred by our respectable dental colleges upon men abroad who have done much to uphold dental science and education? It could not demean a dental school to follow the example of Oxford and Cambridge, or our own Yale and Harvard. The honors might be confined to dentists who have by long service for the profession in dental science, education, and journalism entitled themselves to such consideration. It was a mistake on the part of the National Association of Faculties to put severe restrictions on the schools within its jurisdiction. There are men worthy of honor whose names would honor the schools. This Association has done great service to the profession, and I trust I have written no word of disparagement, and I hope, at least, you will not prevent the subject being discussed.

An American Dentist.

REMARKS.

There are, doubtless, many who feel exactly as our correspondent. He must understand, however, that the Association of Faculties, in prohibiting any college from granting an honorary degree, simply followed a line of practice adopted by all professional schools in this country. This grew out of the fact that professional degrees had been bought and sold and are to-day bought and sold *in ab-*

34

*sentia,* and it required a positive interdiction to prevent the charge being made that the colleges were engaged in a similar traffic.

The subject has been fully threshed out in former years, and it is not at all likely that dental colleges will be permitted to grant these degrees, and it certainly is to be hoped that no effort will be made to revive such a dangerous practice.—ED.

